# Food Approach and Food Avoidance in Young Children: Relation with Reward Sensitivity and Punishment Sensitivity

**DOI:** 10.3389/fpsyg.2016.00928

**Published:** 2016-06-24

**Authors:** Laura Vandeweghe, Leentje Vervoort, Sandra Verbeken, Ellen Moens, Caroline Braet

**Affiliations:** Department of Developmental, Personality and Social Psychology, Ghent UniversityGhent, Belgium

**Keywords:** Eating behavior, Punishment Sensitivity, Reward Sensitivity, Food Approach, Food Avoidance, Preschool children

## Abstract

It has recently been suggested that individual differences in Reward Sensitivity and Punishment Sensitivity may determine how children respond to food. These temperamental traits reflect activity in two basic brain systems that respond to rewarding and punishing stimuli, respectively, with approach and avoidance. Via parent-report questionnaires, we investigate the associations of the general motivational temperamental traits Reward Sensitivity and Punishment Sensitivity with Food Approach and Food Avoidance in 98 preschool children. Consistent with the conceptualization of Reward Sensitivity in terms of approach behavior and Punishment Sensitivity in terms of avoidance behavior, Reward Sensitivity was positively related to Food Approach, while Punishment Sensitivity was positively related to Food Avoidance. Future research should integrate these perspectives (i.e., general temperamental traits Reward Sensitivity and Punishment Sensitivity, and Food Approach and Avoidance) to get a better understanding of eating behavior and related body weight.

## Introduction

Food is a primary reinforcer, shaping behavior through learning processes (Berridge, 1996). Sometimes, food acts as a positive, appetitive reward activating pleasant thoughts and approach behavior (Berridge, 1996; Saper et al., 2002). Sometimes, food acts as a negative, aversive punishment, activating disgust and avoidance behavior (Rozin and Fallon, 1980; Batsell et al., 2002). In the field of eating behavior, different concepts have been used to describe movements toward or away from food. In the current study, eating behaviors and thoughts that involve a movement toward or desire for food are labeled as Food Approach (e.g., overeating, emotional eating, external eating, eating in the absence of hunger, enjoyment of food), while eating behaviors that involve a movement away from food are labeled as Food Avoidance (e.g., food neophobia, picky/fussy eating, slowness in eating, emotional undereating). Since people differ in their response to food, the present study investigates the relation between general individual temperamental traits that may determine one’s susceptibility to the appetitive/rewarding or aversive/punishing properties of food and specific eating-related behaviors.

The difference in how individuals respond to food might depend on their individual trait differences in automatic responding to a broad range of environmental cues, namely Reward Sensitivity (i.e., reward-related approach motivation) and Punishment Sensitivity (i.e., punishment-related avoidance motivation) (Gray, 1994). Reward Sensitivity and Punishment Sensitivity are indeed implicated in eating behavior and eating difficulties (Davis et al., 2004; Bijttebier et al., 2009). Davis et al. (2007) described a model with two pathways linking Reward Sensitivity to eating behavior in a sample of adult women. First, Reward Sensitivity was considered a risk factor for overeating (i.e., binge eating, emotionally driven eating, and external eating). Reward Sensitivity has indeed been found to be related to overeating, with individuals higher in Reward Sensitivity reporting more overeating compared to individuals lower in Reward Sensitivity (Loxton and Dawe, 2001; Davis et al., 2004). Second, Reward Sensitivity was thought to be a determinant of food preferences (Davis et al., 2007). Consistently, adults with high Reward Sensitivity, compared to low Reward Sensitivity, showed a preference for sweet taste (Saliba et al., 2009) and spicy foods (Byrnes and Hayes, 2013). Not only preference for sweet food, but also intake of high energy products has been found to be determined by Reward Sensitivity (De Cock et al., 2015, 2016; De Decker et al., 2016). Daily intake of snacks and sugar sweetened beverages was higher in individuals with higher Reward Sensitivity, especially in adolescent girls (De Cock et al., 2015). Furthermore, Reward Sensitivity has been considered a risk factor for eating disorders (Kane et al., 2004; Claes et al., 2006). Since Reward Sensitivity is related to approach behavior (Gray, 1994), it might be assumed that individuals with eating disorders characterized by a FApB are more sensitive to reward compared to healthy controls (Loxton and Dawe, 2001; Harrison et al., 2010). Consistent with this assumption, patients with eating disorders involving binge eating have been found to be more sensitive to reward compared to healthy controls (Kane et al., 2004; Harrison et al., 2010).

The role of Reward Sensitivity in eating behavior is well described in both adolescents and adults. However, the implications of Punishment Sensitivity in this domain are less studied. Nevertheless, Punishment Sensitivity has also been conceptually and empirically linked to eating disorders (Claes et al., 2006; Beck et al., 2009). Since Punishment Sensitivity is related to inhibition or avoidance behavior (Gray, 1994), it might be assumed that individuals with an eating disorder characterized by food avoidance behavior are more sensitive to punishment compared to healthy controls. Indeed, individuals with eating disorders involving purging or chronic self-starvation have been found to be more sensitive to punishment compared to healthy controls (Claes et al., 2006; Harrison et al., 2010; Matton et al., 2015).

Punishment Sensitivity and Reward Sensitivity are derived from Gray’s Reinforcement Sensitivity Theory (Gray, 1970, 1987b; Gray and McNaughton, 2000). This neuropsychological theory describes two motivational systems that control behavior: the Behavioral Approach System and the Behavioral Inhibition System, respectively, resulting in two dimensional temperamental traits that vary between people: Reward Sensitivity and Punishment Sensitivity. *Reward Sensitivity* is assumed to reflect the sensitivity of BAS, *Punishment Sensitivity* the sensitivity of BIS. The BAS is thought to respond to rewarding environmental stimuli by activation of the dopaminergic system (Gray, 1994; Depue and Collins, 1999) in the brain regions implicated in reward processing (i.e., mesocorticolimbic pathways) (Risinger et al., 2000; Di Chiara et al., 2004). This triggers the initiation of “approach” behavior aimed at obtaining the reward (Kane et al., 2004). People high in Reward Sensitivity are assumed to have a highly sensitive BAS-system, easily activated by reward and exhibiting stronger appetitive responses compared to people low in Reward Sensitivity. Interestingly, the brain does not seem to differentiate whether the reward is provoked by natural reward (e.g., palatable food), behavior (e.g., winning a bet), or pharmacologic agents (e.g., illicit drugs) (Kelley et al., 2005). The BIS is assumed to react on signals of conditioned aversive events (e.g., punishment or non-reward), novelty, and innate fear stimuli. Brain structures considered to be involved in the BIS system are the septohippocampal system and its monoaminergic afferents from the brainstem (Gray, 1994; Gray and McNaughton, 2003). The activation of the BIS in response to these stimuli causes inhibition of ongoing behavior or avoidance of aversive stimuli. People high in Punishment Sensitivity are assumed to have a highly sensitive BIS-system, easily activated when confronted with punishment and exhibiting stronger inhibitory or avoidant responses, compared with people scoring lower on Punishment Sensitivity (Gray, 1978, 1987a, 1990; Carver and White, 1994). Variations in Punishment Sensitivity and Reward Sensitivity can explain individual differences in affectivity, personality, and behavior in different domains of life (Corr, 2004; Bijttebier et al., 2009).

Until now, research investigating the link between Punishment Sensitivity, Reward Sensitivity, and eating behavior has mainly focused on adolescents and adults. This is surprising, as both temperamental differences and eating behaviors develop early, and may track into adolescence and adulthood (Nicklaus and Remy, 2013). Furthermore, eating problems in early childhood (e.g., unpleasantness at meals, struggle over eating, eating little, pickiness, eating slowly, and low interest in food) are common in young children (Marchi and Cohen, 1990; Stice et al., 1999; Jacobi et al., 2003; McDermott et al., 2008) and constitute a risk factor for developing parallel pathological problems in childhood and adolescence (Marchi and Cohen, 1990). Despite the importance of studying eating behaviors in early age, research investigating the link between Punishment Sensitivity and Reward Sensitivity, and eating behavior in this crucial developmental period is scarce. One notable exception is a recent study investigating the effect of Reward Sensitivity and feeding strategies on willingness to taste disliked food items in preschool children (Vandeweghe et al., 2016). Research on the relation between Reward Sensitivity, Punishment Sensitivity, and eating behaviors in preschool children is highly relevant, since knowledge on the determinants of eating behavior in childhood increases the understanding of both normal and problematic eating behavior, and the development of eating disorders later on. The current study therefore examines the relation between Reward Sensitivity, Punishment Sensitivity, Food Approach, and Food Avoidance in young children. We predict that Reward Sensitivity will be positively correlated with Food Approach, while Punishment Sensitivity will be positively correlated with Food Avoidance. The present study is one step in understanding how general basic temperamental traits (i.e., Reward Sensitivity and Punishment Sensitivity) are related to more proximal factors (i.e., specific food approach and avoidance behavior or thoughts) determining eating habits.

## Method

### Participants

In total, 98 mothers (age: *M* = 35.03; *SD* = 4.89) of preschool children (56.1% boys; age: *M* = 4.87; *SD* = 1.13) completed the questionnaires as part of a larger research project. According to the adjusted Body Mass Index (BMI) for children (Actual BMI/Percentile 50 of BMI for age and gender × 100) (Roelants and Hauspie, 2004), children had a mean adjusted BMI of 97.34 (*SD* = 11.60) ranging between 67.38 and 134.28. According to the Highest Household Educational Attainment (HHEA, as a proxy for Socio Economic Status), 18.4% of the households have completed high school and 80.6% have a bachelor’s degree or higher. Data on HHEA were missing in 1% of the cases.

### Procedure

Participants were recruited by 3rd-year psychology students of Ghent University as partial fulfillment of course requirements. Each student had to find two families with a preschool child (via relatives, friends, acquaintances, school,..) that were willing to participate. The students were thoroughly informed about the content of the questionnaires and trained to administer them. They were instructed to visit the participants at home, administer the questionnaire in a quiet place, and be available when questions arise. Active informed consent was obtained from each mother prior to completing the questionnaires. The study procedure was approved by the Institutional Ethical Committee.

## Materials

### Punishment Sensitivity and Reward Sensitivity

#### Behavioral Inhibition System/Behavioral Approach System (BIS/BAS) Scales

The Dutch parent version of the BIS/BAS-scales (Vervoort et al., 2015) is based on an age-downward adaptation (Muris et al., 2005) of the original self-report scales for adults (Carver and White, 1994). Twenty items are scored on a 4-point Likert Scale from 1 (not true) to 4 (very true), with higher scores indicating higher Punishment Sensitivity and Reward Sensitivity. Punishment Sensitivity is measured with the BIS-scale (BIS_Total, 7 items), which includes statements such as “My child is very fearful compared to his/her friends”. Reward Sensitivity is measured with the BAS-scale (BAS_Total, 13 items) and can be further subdivided in 3 subscales. The Reward Responsiveness subscale (BAS_RR, 5 items) includes statements such as “It would excite my child to win a contest”. The Fun Seeking subscale (BAS_FS, 4 items) includes statements such as “My child craves excitement and new sensation”. The Drive subscale (BAS_D, 4 items) includes statements such as “When my child wants something, he or she usually goes all out to get it”. The BIS and BAS scales of the Dutch BIS/BAS parent version have meaningful relations with other Punishment Sensitivity/Reward Sensitivity instruments (Vervoort et al., 2015). Internal consistency in the present sample was good for the BAS_Total scale (Cronbach’s α = 0.85) and BAS_D subscale (Cronbach’s α = 0.84); acceptable for BAS_RR subscale (Cronbach’s α = 0.74) and BIS_Total scale (Cronbach’s α = 0.77), but poor for BAS_FS subscale (Cronbach’s α = 0.46). Similar to previous studies excluding scales with low internal consistency (e.g., Horan et al., 2006; Buchholz et al., 2007; Scott et al., 2015), the BAS_FS subscale was not included in the analyses.

#### Sensitivity to Punishment and Sensitivity to Reward Questionnaire (SPSRQ)

The SPSRQ is a Dutch parent report questionnaire (Luman et al., 2012), measuring Punishment Sensitivity and Reward Sensitivity (Colder and O’connor, 2004). The SPSRQ consists of 33 items, scored on a 5-point Likert Scale from 1 (strongly disagree) to 5 (strongly agree), with higher scores indicating higher Punishment Sensitivity and Reward Sensitivity. The items can be divided in two scales: a Punishment Sensitivity scale (SPSRQ-PS; 15 items) and a Reward Sensitivity scale (SPSRQ-RS; 18 items). SPSRQ-PS includes statements such as “Your child is a shy person”. SPSRQ-RS includes statements such as “Your child does a lot of things for approval”. The Dutch parent report version of the SPSRQ was found to be a valid instrument to assess Reward and Punishment Sensitivity in children (Luman et al., 2012). Internal consistency in the present sample was good for both the SPSRQ-PS scale (Cronbach’s α = 0.81) and the SPSRQ-RS scale (Cronbach’s α = 0.84).

### Food Approach and Food Avoidance

#### Child Food Neophobia Scale

The CFNS (Pliner, 1994) assesses the extent to which children reject novel or unknown foods (i.e., Food Avoidance) and originally consists of 10 items, including statements such as “My child does not trust new foods”. The items are scored on a 4-point Likert Scale from 1 (strongly disagree) to 4 (strongly agree). Higher scores indicate a stronger display of food neophobia. We used the 6-item version that is more adapted to the age range of our sample (Cooke et al., 2003, 2004). The Dutch 6-item version (Goossens and Braet, 2012) has shown good convergent validity (Vandeweghe et al., 2016). Internal consistency in the present sample was good (Cronbach’s α = 0.89).

#### Child Eating Behavior Questionnaire (CEBQ)

The Dutch version of the CEBQ (Sleddens et al., 2008) is a 35- item parent-report questionnaire that assesses eating-approach as well as eating-avoidance behaviors, scored on a 5-point Likert Scale from 1 (never) to 5 (always), with higher scores indicating a stronger display of food approach or food avoidance behavior. Food Approach Behavior (FApB, 16 items) is measured with 4 subscales: Food Responsiveness (FR, 5 items), Desire to Drink (DD, 3 items), Emotional Over-Eating (EOE, 4 items) and Enjoyment of Food (EF, 4 items). Food Avoidance Behavior (FAvB, 19 items) is measured with 4 subscales: Satiety Responsiveness (SaR, 5 items), Food Fussiness (FF, 6 items), Slowness in Eating (SE, 4 items), and Emotional Under-Eating (EUE, 4 items) (Wardle et al., 2001). The questionnaire is found to be a psychometrically sound tool to measure these eating behaviors (Sleddens et al., 2008). Internal consistency in the present sample was excellent for FF (Cronbach’s α = 0.94); good for FAvB (Cronbach’s α = 0.89), EF (Cronbach’s α = 0.89), EUE (Cronbach’s α = 0.81); acceptable for FApB (Cronbach’s α = 0.79), SaR (Cronbach’s α = 0.76), FR (Cronbach’s α = 0.74); and questionable for SE (Cronbach’s α = 0.69) and EOE (Cronbach’s α = 0.63).

#### Dutch Eating Behavior Questionnaire (DEBQ)

A parent version of the DEBQ (Braet and Van Strien, 1997) was used to measure two different types of eating behavior: emotional eating and external eating. Both behaviors can be seen as a Food Approach. The emotional eating scale (EMO, 13 items) includes statements such as “Does your child feel like having food when he/she feels restless?” and the external eating scale (EXT, 10 items) includes statements such as “When your child sees or smells delicious food, does he/she feels like having some?”. The items are scored on a 5-point Likert Scale from 1 (never) to 5 (very often) with higher scores indicating more external eating. Studies on the parent version of the DEBQ (Braet and Van Strien, 1997; Braet et al., 2007) revealed convergent validity and sufficient internal consistency. Internal consistency in the present sample was excellent for EMO (Cronbach’s α = 0.93) and good for EXT (Cronbach’s α = 0.82).

#### Power of Food Scale (PFS)

The Dutch parent version of the PFS (Verbeken and Braet, 2014) is based on an age-downward adaptation (Lowe, 2006) of the original self-report scales for adults (Cappelleri et al., 2009). The scale (PFS, 15 items) assesses the appetitive responsiveness to today’s food-abundant environment by means of appetite-related thoughts, feelings and motivations, and is, therefore, a proxy for Food Approach. The three subscales within the questionnaire differentiate between three levels of food proximity: (1) when food is readily available but not present (AnP, 4 items), (2) when food is present, but not tasted (PnT, 5 items) and (3) when food is tasted, but not eaten (TnE, 6 items). The items are scored on a 5-point Likert Scale from 1 (I don’t agree at all) to 5 (I strongly agree) with higher scores indicating greater appetitive responsiveness to rewarding properties of the food environment. AnP includes statements such as “My child thinks about food even when he/she is not truly hungry.”, PnT includes statements such as “If my child sees or smells a food it likes, he/she gets a very strong desire to have some” and TnE includes statements such as “My child enjoys eating a lot more than most other kids”. Internal consistency in the present sample was good for PFS (Cronbach’s α = 0.86) and acceptable for AnP (Cronbach’s α = 0.74), PnT (Cronbach’s α = 0.71) and TnE (Cronbach’s α = 0.75).

### Data Analytical Plan

The Missing Completely At Random test (MCAR; Little, 1988) was performed to determine whether the missing values were likely to be missing at random. If *p* > 0.05 for the normed χ^2^ test statistic, values will be imputed for the missing data, following the Expectation Maximization algorithm available in SPSS (Schafer, 1997).

Then, correlations were calculated between measures of Reward and Punishment Sensitivity, and Food Approach and Food Avoidance. Even if the scales are not normally distributed, we can proceed with deviations from normality because of the central limit theorem (Field, 2009). As our sample is fairly big (*n* = 98), Pearson’s *r* was used to examine the correlations between the measures of Reward Sensitivity and the Food Approach scales of the CEBQ, DEBQ, and PFS; and the measures of Punishment Sensitivity and the Food Avoidance scales of the CEBQ and CFNS. Effect sizes of these associations were evaluated as small if *r* = 0.10, medium if *r* = 0.30 and large if *r* = 0.50 (Cohen, 1977).

In order to assess the relative importance of Reward Sensitivity and Punishment Sensitivity for Food Approach and Food Avoidance, two multiple linear regression analyses were conducted. Four principal component analyses were first performed to compute a component score for Reward Sensitivity, Punishment Sensitivity, Food Approach and Food Avoidance. The component scores of Reward Sensitivity, Punishment Sensitivity, Food Approach and Food Avoidance were then used as variables in the multiple regression analyses. Two independent hierarchical regression analyses for each of the two components Food Approach and Food Avoidance were conducted. Control variables Age, Sex and adjusted BMI as well as one of the two predictors (i.e., Reward Sensitivity or Punishment Sensitivity) were entered in the first step of the analyses, while the other predictor was entered in the second step. The explained variance was considered small if *R*^2^ = 1%, moderate if *R*^2^ = 9% and large if *R*^2^ = 25% (Cohen, 1977). The present study, with 98 participants, α = 0.05 and β = 0.20, was sufficiently powered to detect small effects.

## Results

### Missing Values

In total, 81 mothers filled out the questionnaires completely, resulting in 20 of 8232 missing data points (0.2% of the data). A normed χ^2^ of 1, *p* = 0.45 (χ^2^ = 1149.26/df = 1145) indicated that missing values were missing completely at random (MCAR; Little, 1988). Following the Expectation Maximization algorithm, values for the missing data were imputed (Schafer, 1997).

### Correlations

The correlation matrix and descriptive statistics of the study variables are depicted in **Table 1**.

**Table 1:** Descriptive statistics and correlations (Pearson’s r) between Reward Sensitivity, Punishment Sensitivity, Food Approach, and Food Avoidance in preschoolers.

					RS-indices				PS-indices	
					BAS_Total	BAS_D	BAS_RR	SPSRQ-RS	BIS_Total	SPSRQ-PS
						
			*M (SD)*		33.19 (6.32)	9.30 (2.88)	14.79 (2.52)	52.12 (8.67)	15.36 (3.71)	33.48 (8.99)
				Range	34	12	12	50	17	59
Food Approach	CEBQ	FApB	38.42 (6.97)	39	0.30^∗∗^	0.22^∗^	0.32^∗∗^	0.35^∗∗^	0.08	-0.03
		EF	13.42 (2.97)	15	0.14	0.07	0.23^∗^	0.04	-0.07	-0.11
		FR	10.62 (3.25)	17	0.26^∗∗^	0.21^∗^	0.26^∗∗^	0.44^∗∗^	0.07	0.05
		EOE	6.66 (2.04)	8	0.03	0.13	-0.03	0.30^∗∗^	0.26^∗∗^	0.17
		DD	7.84 (2.69)	12	0.25^∗^	0.11	0.24^∗^	0.10	0.02	-0.15
	DEBQ	EMO	21.14 (7.20)	26	0.00	0.05	0.00	0.20^∗^	0.24^∗^	0.21^∗^
		EXT	30.58 (5.16)	25	0.29^∗∗^	0.31^∗∗^	0.25^∗∗^	0.46^∗∗^	0.10	0.06
	PFS	PFS•	32.06 (9.47)	38	0.36^∗∗^	0.34^∗∗^	0.35^∗∗^	0.40^∗∗^	0.20^∗^	0.11
		AnP	12.32 (4.17)	18	0.23^∗^	0.21^∗^	0.25^∗^	0.29^∗∗^	0.22^∗^	0.12
		PnT	12.96 (3.84)	16	0.33^∗∗^	0.31^∗∗^	0.32^∗∗^	0.35^∗∗^	0.17	0.08
		TnE	6.72 (2.93)	13	0.35^∗∗^	0.32^∗∗^	0.32^∗∗^	0.37^∗∗^	0.11	0.06

Food Avoidance	CEBQ	FAvB	54.66 (11.03)	58	0.03	0.04	0.03	0.08	0.21^∗^	0.23^∗^
		SaR	14.88 (3.12)	14	0.05	0.06	0.05	0.08	0.26^∗∗^	0.15
		SE	11.64 (2.69)	14	-0.07	0.00	-0.10	0.01	0.20^∗^	0.05
		EUE	10.75 (3.38)	16	0.12	0.13	0.12	0.17	0.18	0.11
		FF	17.39 (5.76)	23	-0.00	-0.03	0.01	0.01	0.05	0.27^∗∗^
	CFNS		13.86 (4.11)	18	-0.01	0.01	-0.02	0.02	0.08	0.24^∗^

*^∗^*p* < 0.05, ^∗∗^*p* < 0.01.*

*•Composite score of the Power of Food Scale.*

*CEBQ, Child Eating Behavior Questionnaire; DEBQ, Dutch Eating Behavior Questionnaire; PFS, Power of Food Scale; CFNS, Child Food Neophobia Scale; FApB, Food Approach Behavior; EF, Enjoyment of Food; FR, Food Responsiveness; EOE, Emotional Overeating; DD, Desire To Drink; EMO, Emotional Eating; EXT, External Eating; AnP, Available but not Present; PnT, Present but not Tasted; TnE, Tasted but not Eaten; FAvB, Food AvoidanceBehavior; SaR, Satiety Responsiveness; SE, Slowness in Eating; EUE, Emotional Undereating; FF, Food Fussiness; RS, Reward Sensitivity; PS, Punishment Sensitivity; BAS, BehavioralApproach System; BAS_D, Drive; BAS_RR, Reward Responsiveness; SPSRQ, Sensitivity to Punishment and Sensitivity to Reward Questionnaire; BIS, Behavioral Inhibition System.*

#### Food Approach

Concerning the CEBQ scales measuring Food Approach, BAS_Total and BAS_D correlated significantly positively with FApB and Food Responsiveness, but not with Enjoyment of Food, and Emotional Overeating. BAS_RR correlated significantly positively with FApB, Enjoyment of Food, Food Responsiveness, but not with Emotional Overeating. SPSRQ-RS positively correlated with FApB, Food Responsiveness, and Emotional Overeating, but nut with Enjoyment of Food. All Reward Sensitivity indices correlated significantly positively with all PFS scales and External Eating. Only SPSRQ-RS, but not the other Reward Sensitivity indices, correlated significantly positively with Emotional Eating. The significant correlations between Reward Sensitivity indices and Food Approach scales were small to large with effect sizes ranging from 0.20 to 0.46. Furthermore, BIS_Total correlated significantly positively with Emotional Overeating, Emotional Eating, PFS, and AnP. SPSRQ-PS was significantly positively correlated with Emotional Eating. The significant correlations between Punishment Sensitivity indices and scales measuring Food Approach were small to moderate with effect sizes ranging from 0.20 to 0.26. Besides these scales, no other scales measuring Food Approach were correlated with Punishment Sensitivity indices.

#### Food Avoidance

Both Punishment Sensitivity indices significantly positively correlated with FAvB. Furthermore, BIS_Total significantly positively correlated with Satiety Responsiveness and Slowness in Eating, but not with Emotional Undereating, Food Fussiness, and CFNS. SPSRQ-PS correlated significantly positively with Food Fussiness and CFNS, but not with Satiety Responsiveness, Slowness in Eating, and Emotional Undereating. The significant correlations between Punishment Sensitivity indices and Food Avoidance scales were small to moderate with effect sizes ranging from 0.20 to 0.27. None of the Reward Sensitivity indices correlated significantly with any of the scales measuring Food Avoidance.

### Regression Analyses

First, four principal component analyses were performed to compute a component score for Reward Sensitivity, Punishment Sensitivity, Food Approach, and Food Avoidance. A first principal component analysis on BAS_Total and SPSRQ-RS resulted in loadings of 0.90 on the component Reward Sensitivity, explaining 81.05% of the variance. A second principal component analysis on BIS_Total and SPSRQ-PS resulted in loadings of 0.87 on the component Punishment Sensitivity, explaining 77.15% of the variance. A third principal component analysis on FApB, External Eating and Emotional Eating (of the DEBQ) and PFS resulted in loadings of >0.68 on the component Food Approach, explaining 63.44% of the variance. A fourth principal component analysis on FAvB and CFNS resulted in loadings of >0.93 on the component Food Avoidance, explaining 88.40% of the variance. The component scores of Reward Sensitivity, Punishment Sensitivity, Food Approach and Food Avoidance were used as variables in the multiple regression analyses.

**FIGURE 1:**
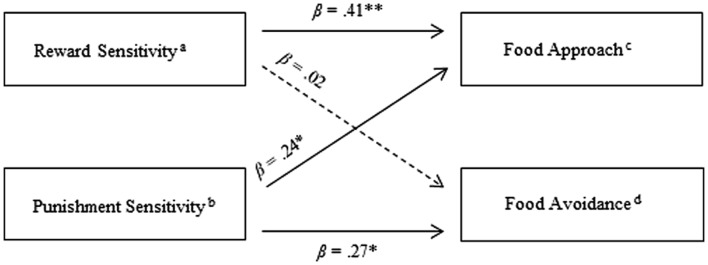
**Summary of independent hierarchical regression analyses for Reward and Punishment Sensitivity predicting Food Approach and Food Avoidance**. ^∗^*p* < 0.05, ^∗∗^*p* < 0.01; ^a^ Component score of BAS_Total and SPSRQ-RS; ^b^ Component score of BIS_Total and SPSRQ-PS; ^c^ Component score of FApB, External Eating, Emotional Eating and the Power of Food Scale; ^d^ Component score of FAvB and CFNS.

Second, two independent hierarchical regression analyses for each of the two components Food Approach and Food Avoidance were conducted. Control variables Age, Sex, adjusted BMI as well as one of the two predictors (i.e., Reward Sensitivity or Punishment Sensitivity) were entered in the first step of the analyses, while the other predictor was entered in the second step. Standardized betas (β) revealed that Reward Sensitivity (β = 0.41, *p* < 0.001) and Punishment Sensitivity (β = 0.24, *p* = 0.01) were significantly related to Food Approach while Punishment Sensitivity (β = 0.27, *p* = 0.01) and not Reward Sensitivity (β = 0.02, *p* = 0.84) were significantly related to Food Avoidance (see **Figure 1**). After controlling for Age, Sex, adjusted BMI and Punishment Sensitivity, Reward Sensitivity explained 16.8% of the variance in Food Approach [*F* change (1.92) = 20.50, *p* < 0.001], indicating a moderate to large effect. After controlling for Age, Sex, adjusted BMI and Reward Sensitivity, Punishment Sensitivity explained 6.5% of the variance in Food Avoidance [*F* change (1.92) = 6.46, *p* = 0.01], indicating a small to moderate effect.

## Discussion

We investigated the association between the general temperamental traits Reward and Punishment Sensitivity, and the more specific eating related concepts Food Approach and Avoidance in a large sample of healthy preschool children. Our results largely confirmed our hypotheses that Reward Sensitivity and Punishment Sensitivity are positively linked with Food Approach and Food Avoidance, respectively, although some surprising findings emerged.

### Reward Sensitivity and Food Approach

As expected, the temperamental trait Reward Sensitivity is implicated in Food Approach. All Reward Sensitivity indices were significantly positively correlated with the combined CEBQ subscales measuring food approach behavior (i.e., FApB). By looking more closely at the different Reward Sensitivity scales and the different FApB subscales, we can unravel which specific aspects of Reward Sensitivity are embedded in each of the specific food approach behaviors. Reward Responsiveness (i.e., BAS_RR) was positively related with Enjoyment of Food and Food Responsiveness, but not with Emotional Overeating. The items of Enjoyment of Food (e.g., “my child loves food”, “my child enjoys eating”) and Food Responsiveness (e.g., “My child is always asking for food”, “If allowed to, my child would eat too much”) cover indeed the anticipation or reaction on (food) reward, which is consistent with the conceptualization of BAS_RR as described by Carver and White (1994). The unexpected null-finding for Emotional Overeating can be explained by the content of the items; they do not contain this anticipation, but rather refer to an emotion regulation strategy in which eating is a reaction to bad feelings. Furthermore, SPSRQ-RS was positively related with Food Responsiveness and Emotional Overeating, but surprisingly not with Enjoyment of Food. We assume that these findings might be due to the different levels of generalization in the scales: Enjoyment of Food items refer to general situations, while SPSRQ-RS (and Food Responsiveness and Emotional Overeating) refer to more specific concrete situations (e.g., SPSRQ-RS: “your child enjoys being the center of attention”; Food Responsiveness: “My child is always asking for food”; Emotional Overeating: “My child eats more when annoyed”).

The BAS_D subscale was significantly correlated with Food Responsiveness, but contrary to our expectations, not with the other Food Approach scales of the CEBQ. Moreover, the significant effect was small. These findings might suggest that the general drive component of Reward Sensitivity is less captured by these specific Food Approach indices. This is unfortunate, because this particular aspect of Reward Sensitivity (i.e., persistent pursuit of desired goals) has repeatedly been found to be an important determinant of eating behavior and overweight (Dawe and Loxton, 2004; Verbeken et al., 2012). Thus, given that the drive component is important, it might be valuable to measure it when assessing eating behavior. Therefore, it might be valuable to add a drive component to the CEBQ including items such as “My child would do anything to get the food (s)he wants”.

Conform our hypotheses, all Reward Sensitivity indices were positively related to the External Eating scale of the DEBQ, which suggests that children with higher Reward Sensitivity, compared to lower Reward Sensitivity, are more susceptible to eating based on external stimuli (i.e., hedonic eating) rather than on internal homeostatic signals. Furthermore, all Reward Sensitivity indices were positively related to all scales of the Power of Food Scale (i.e., the subscales as well as the total score). These findings support the idea that the Power of Food Scale can be used as a valid index of a child’s specific sensitivity to the rewarding value of food (Lowe and Butryn, 2007). Children with higher trait Reward Sensitivity in general, seem to be highly sensitive to food reward, not only when food is tasted but not eaten (i.e., TnE) or present but not tasted (i.e., PnT), but also when food is available but not present (i.e., AnP). As such, high reward sensitive children, characterized by these appetite-related motives, might be more prone to maladaptive eating behavior, such as overeating or eating in absence of hunger, compared to low reward sensitive children.

Unexpectedly, sensitivity to food reward (as measured with the Power of Food Scale) was also determined by Punishment Sensitivity, as shown by the significant positive correlations of BIS_Total with the Power of Food Scale (i.e., composite score of the subscales) and AnP. This might be explained by the semantics of the items; while items of PnT and TnE clearly indicate that food is seen as a reward, items of the AnP are largely about how preoccupied the child is with food (e.g., “My child thinks about food, even when (s)he is not physically hunger”). These items refer to the general motivational salience of food, irrespective of its rewarding or punishing character. Unlike the unambiguous PnT and TnE items, the items of AnP can be understood in two ways: the child can think about food because (s)he wants it and (s)he sees food as a reward, or the child can think about food in a negative way. We assume that children having a negative relation with food, such as food neophobics or food restrictive children, might also be preoccupied or controlled by food (e.g., “It seems like food controls my child rather than the other way around”), but in a negative way.

### Punishment Sensitivity and Food Avoidance

As expected, Punishment Sensitivity is implicated in Food Avoidance. Most Punishment Sensitivity indices were significantly positively correlated with the Food Avoidance scales of the CEBQ and with the CFNS. More specifically, the combined CEBQ subscales measuring food avoidance behavior (i.e., FApB) were significantly positively related to both Punishment Sensitivity indices (i.e., BIS_Total and SPSRQ-PS). Noteworthy, the other Food Avoidance scales were related to either BIS_Total or to SPSRQ-PS, but not to both: Food Fussiness and Food Neophobia (indexed by CNFS) were only related to SPSRQ-PS, while Satiety Responsiveness and Slowness in Eating were only related to BIS_Total. Almost all items of Food Fussiness and CFNS reflect fear for unknown or new food items (e.g., Food Fussiness: “My child refuses new foods at first”; CFNS: “My child does not trust new foods”), which is captured by SPSRQ-PS, and not BIS_Total, as the former but not the latter includes items regarding fear for novelty (e.g., “My child is afraid of new or unexpected situations”). Items of Satiety Responsiveness and Slowness in Eating do not contain this novelty-factor; they rather refer to hunger and satiety. The responses on hunger and satiety are possibly influenced by stress and fear in general, which is rather captured by BIS than by SPSRQ-PS.

### Punishment Sensitivity and Food Approach

Our hypotheses that Punishment Sensitivity and Reward Sensitivity are associated with Food Avoidance and Approach, respectively, are based on Gray’s Reinforcement Sensitivity Theory, linking Punishment Sensitivity with avoidance and Reward Sensitivity with approach (Gray, 1994). Therefore, we did not formulate hypotheses concerning Punishment Sensitivity and Food Approach. However, exploratively, we found that Punishment Sensitivity might also be a determinant of approach behavior, as suggested by the significant correlation between Punishment Sensitivity and Food Approach in the regression analyses. This finding might be related to a specific kind of approach behavior, namely (over)eating as a reaction to emotional situations, given the significant positive correlations between Punishment Sensitivity indices and Emotional Overeating and Emotional Eating. In such emotional situations, food can be seen as a way of coping with negative emotions (e.g., Herman and Polivy, 1988; Heatherton and Baumeister, 1991); in other words, a means to reach a goal (i.e., comfort) instead of the goal itself. The link between Punishment Sensitivity and emotional eating in response to negative events is consistent with the findings that Punishment Sensitivity is strongly associated with negative affect (Watson et al., 1999).

### Limitations and Future Research

Based on the theoretical background of Reward Sensitivity and Punishment Sensitivity, we might assume that Reward Sensitivity and Punishment Sensitivity determine the extent to which someone approaches or avoids food and not the other way around. However, due to the cross-sectional nature of the results, we cannot be certain of the direction of the associations. Furthermore, since the variables were assessed concurrently, a longitudinal design might be interesting to get more insight in the developmental course of Food Approach or Avoidance and the link with temperament. Next, the current study was conducted in a convenient sample. It might also be interesting to investigate the role of temperamental traits in children with severe eating problems. In the present study, we used parent report instruments to assess the concepts of Reward Sensitivity, Punishment Sensitivity, Food Approach and Food Avoidance. Validity of these instruments is well-established in Dutch speaking samples except for the Power of Food Scale for which validity reports in this population are lacking. Furthermore, although parent report is found to be a valid method for child temperament assessment (Copeland et al., 2004; Vervoort et al., 2015), it would be valuable to replicate the current findings with behavioral measures of Food Approach and Food Avoidance.

In spite of finding evidence for a positive relation between Reward Sensitivity and Food Approach, and Punishment Sensitivity and Food Avoidance, the explained variance was at best moderate (Cohen, 1977). The effect sizes are slightly smaller compared to other studies in this age group (De Pauw et al., 2009; Vervoort et al., 2015). These results suggest that, in addition to Reward Sensitivity and Punishment Sensitivity, other factors may determine whether certain appetite related behaviors or thoughts will be present. This is consistent with biopsychosocial models describing multiple determinants of eating behavior (e.g., Davison and Birch, 2001), such as learning processes, parental feeding styles and the obesogenic environment (e.g., Eertmans et al., 2001; Savage et al., 2007; Scaglioni et al., 2011). Moreover, while not all Reward Sensitivity indices were systematically correlated with CEBQ Food Approach scales, they were so with all scales of the Power of Food Scale. Different from the other Food Approach scales, the Power of Food Scale measures appetite-related thoughts, motivations and feelings instead of actual food (over)consumption. Since thoughts and motives are less controllable and more automatically generated than behavior (Ajzen, 1985), it might be assumed that they are more directly linked to basic bottom-up personality factors like Punishment Sensitivity and Reward Sensitivity, and less influenced by top-down controlled processes (Appelhans, 2009). It would be interesting to investigate whether the thoughts and motives moderate the relation between Reward Sensitivity and actual food consumption, for example in an ad libitum taste test. It might further be interesting to investigate whether Food Approach mediates the relation between Reward Sensitivity and overweight, and as such replicating the full model of Davis et al. (2007) in preschool children.

## Conclusion

Gray’s Reinforcement Sensitivity Theory assumes individual differences in the sensitivity of two basic brain systems that are supposed to react on reward and punishment, being Reward and Punishment Sensitivity. In the current study, these differences are found to have relevance for eating behavior and thoughts in preschool children. This insight substantially enhances our understandings of eating behavior in children. The current study is one step in understanding how basic temperamental traits, such as Reward Sensitivity and Punishment Sensitivity, have an influence on more proximal eating behaviors (i.e., specific food approach and avoidance behavior or thoughts) determining eating habits. We are convinced that, consistent with a biopsychosocial framework, studies investigating eating behavior should not focus on proximal factors alone, but instead combine multiple perspectives to examine how the interactions between these proximal behaviors and distal factors (such as child characteristics) contribute to adaptive and maladaptive eating behaviors.

## Author Contributions

Data was collected by LVDW. LVDW and LV analyzed the data. LVDW drafted the manuscript with substantial input about interpretation of the results and critical revisions provided by all authors. All authors read and approved the final version of the manuscript.

## Conflict of Interest Statement

The authors declare that the research was conducted in the absence of any commercial or financial relationships that could be construed as a potential conflict of interest.

## ABBREVIATIONS

AnP: Available but not Present
BAS: Behavioral Approach System
BAS_D: Drive
BAS_RR: Reward Responsiveness
BIS: Behavioral Inhibition System
CEBQ: Child Eating Behavior Questionnaire
CFNS: Child Food Neophobia Scale
DD: Desire To Drink
DEBQ: Dutch Eating Behavior Questionnaire
EF: Enjoyment of Food
EMO: Emotional Eating
EOE: Emotional Overeating
EUE: Emotional Undereating
EXT: External Eating
FApB: Food Approach Behavior
FAvB: Food Avoidance Behavior
FF: Food Fussiness
FR: Food Responsiveness
PFS: Power of Food Scale
PnT: Present but not Tasted
PS: Punishment Sensitivity
RS: Reward Sensitivity
SaR: Satiety Responsiveness
SPSRQ: Sensitivity to Punishment and Sensitivity to Reward Questionnaire
SE: Slowness in Eating
TnE: Tasted but not Eaten
